# Prevalence of autism spectrum disorder and autistic traits in children with anorexia nervosa and avoidant/restrictive food intake disorder

**DOI:** 10.1186/s13030-021-00212-3

**Published:** 2021-05-17

**Authors:** Takeshi Inoue, Ryoko Otani, Toshiyuki Iguchi, Ryuta Ishii, Soh Uchida, Ayumi Okada, Shinji Kitayama, Kenshi Koyanagi, Yuki Suzuki, Yuichi Suzuki, Yoshino Sumi, Shizuo Takamiya, Yasuko Tsurumaru, Shinichiro Nagamitsu, Yoshimitsu Fukai, Chikako Fujii, Michiko Matsuoka, Junpei Iwanami, Akio Wakabayashi, Ryoichi Sakuta

**Affiliations:** 1 Dokkyo Medical University Saitama Medical Center, Child Development and Psychosomatic Medicine Center, 2-1-50, Minami-Koshigaya, Koshigaya-shi, Saitama-Ken 343-8555 Japan; 2 Department of Pediatrics, Dokkyo Medical University Saitama Medical Center, Saitama, Japan; 3 Department of Pediatrics, Hoshigaoka Maternity Hospital, Aichi, Japan; 4 Department of Pediatrics and Child health, Kurume University School of Medicine, Fukuoka, Japan; 5 Department of Pediatrics, Tachikawa Hospital, Tokyo, Japan; 6 Department of Pediatrics, Okayama University Graduate School of Medicine, Dentistry and Pharmaceutical Sciences, Okayama, Japan; 7 Himeji City Center for the Disabled, Hyogo, Japan; 8 Nagasaki Prefectural Center of Medicine and Welfare for Children, Nagasaki, Japan; 9 Department of Pediatrics, National Hospital Organization Mie National Hospital, Mie, Japan; 10 Department of Pediatrics, Fukushima Medical University School of Medicine, Fukushima, Japan; 11 Mental and developmental clinic for children “Elm Tree”, Hokaido, Japan; 12 Psychiatry Department, Kobe City Nishi-Kobe Medical Center, Hyogo, Japan; 13 Takamiya Psychiatry Clinic, Hyogo, Japan; 14 Department of Pediatrics, Okayama University Hospital, Okayama, Japan; 15 Tokyo Metropolitan Children’s Medical Center, Psychosomatic Medicine, Tokyo, Japan; 16 Department of Neuropsychiatry, Kurume University School of Medicine, Fukuoka, Japan; 17 Department of Psychology, Chiba University, Chiba, Japan

**Keywords:** Autism, Feed and eating disorders, Comorbidity, Anorexia

## Abstract

**Background:**

Autism spectrum disorder (ASD) and feeding and eating disorders (FEDs) such as anorexia nervosa (AN) are strongly linked as evidenced by frequent comorbidity and overlapping traits. However, eating and social behaviors are shaped by culture, so it is critical to examine these associations in different populations. Moreover, FEDs are heterogeneous, and there has been no examination of autistic traits in avoidant/restrictive food intake disorder (ARFID).

**Methods:**

Therefore, we investigated the prevalence of ASD and autistic traits among Japanese children with AN (*n* = 92) or ARFID (*n* = 32) from a prospective multicenter cohort study using the Autism Spectrum Quotient Children’s version (AQC) and Children’s Eating Attitudes Test (ChEAT26).

**Results:**

ASD prevalence was high in both AN and ARFID (16.3 and 12.5%, respectively). The AN group exhibited significantly higher scores on all AQC subscales than an age-matched healthy control (HC) group, but there were no significant correlations between AQC scores and ChEAT26 scores. In the AFRID group, AQC scores did not differ from HCs, but significant correlations were found between total AQC and ChEAT26 scores and between several AQC and ChEAT26 subscales.

**Conclusions:**

Both the AN and ARFID groups had high prevalence rates of ASD. The AN group showed a significantly higher degree of autistic traits than the HC group; however, no difference was found between the ARFID and HC groups. Clinicians need to be aware of these rates when working with children with ED.

**Supplementary Information:**

The online version contains supplementary material available at 10.1186/s13030-021-00212-3.

## Introduction

Feeding and eating disorders (FEDs) are a heterogeneous group of diseases, but all are characterized by persistent pathological food consumption patterns that negatively affect health, emotion, cognition, and quality of life. The most common eating disorders (EDs) in young adults are anorexia nervosa (AN), bulimia nervosa (BN), and binge-eating disorder (BED) [1], and AN and avoidant/restrictive food intake disorder (ARFID) are the most frequent in adolescents [2, 3]. In young females, ED prevalence is especially high, ranging from 0.3 to 1% of the total population [4].

AN, characterized by distorted body image, self-induced starvation, and excessive weight loss with a pathological fear of fat [5], is the most extensively studied FED. Psychosocial, genetic, and cognitive deficits contribute to the onset and maintenance of AN. In addition, there is now compelling evidence that FEDs are associated with autism spectrum disorder (ASD) as originally proposed by Gillberg (1983), as these two disorders are frequently comorbid and exhibit similar cognitive and behavioral features [6], such as restricted and ritual behaviors [7]. ASD is a pervasive developmental disorder characterized by difficulties with social interactions as well as repetitive and restricted behaviors [5]. Although ASDs exist across all cultures and regions, autistic symptoms seem to be vulnerable to the culture in which one is acculturated [8]. Common cognitive processing dysfunctions between ASD and FEDs include an underdeveloped theory of mind [9, 10], lack of empathy [11, 12], alexithymia [13, 14], poor facial recognition [15, 16], limited central coherence [17], set shifting [18], and cognitive inflexibility [19–21]. To understand similar behaviors and common cognitive dysfunctions between ASD and FEDs may lead to enhanced treatment efficacy for ASD and FEDs. On the basis of these shared features, numerous studies over the past several decades have investigated the co-occurrence of ASD or autistic traits in AN. According to a recent systematic review by Huke et al. (2013), ASD prevalence in AN ranges from 8 to 37%, with an average prevalence of 22.9% [22], dramatically higher than the reported ASD prevalence of roughly 1% in the general population [23–25].

Moreover, autistic traits appear more common or severe in AN. Hambrook et al. (2008) first reported that adults with AN showed significantly more numerous and severe autistic traits than matched healthy controls (HCs) using the Autism Spectrum Quotient (AQ) [26], a self-report screening instrument for assessing traits associated with the autistic spectrum in adults originally developed in 2001 by Baron-Cohen et al. [27]. A recent systematic review by Westwood et al. (2016) revealed significantly higher total AQ and AQ Children’s version (AQC) scores in AN, along with significantly higher scores in four of the five AQ/AQC subscales, namely, social skills, attention switching, communication, and imagination [28].AN has higher co-occurrence of ASD and higher autistic traits compared to HC; however, to our knowledge, there are no comparable studies from Asian countries, therefore, it is vital to investigate the prevalence of ASD and autistic traits in AN in a different culture (Japan). Although the development and maintenance of FEDs and the difficulties and challenges faced by children with ASD are heavily influenced by cultural beliefs and attitudes [29, 30], we hypothesize that there is a high prevalence of ASD and autistic traits in AN in Japan as well.

During the period covered by the Diagnostic and Statistical Manual of Mental Disorders-Fourth Edition (DSM- IV-TR, 2000–2013), over 50% of children and adolescents with ED met the criteria for Eating Disorder Not Otherwise Specified, which hampered timely diagnosis and proper treatment [31, 32]. Therefore, Lask and colleague developed the Great Ormond Street criteria to describe a range of eating difficulties in children, including AN, BN, food avoidance emotional disorder, selective eating, functional dysphagia, and pervasive refusal syndrome [31]. ARFID was newly classified in the FEDs section of the DSM-5 (American Psychiatric Association & American Psychiatric Association DSM-5 Task Force). It is characterized by low nutritional state and limited food consumption not associated with body image distortion [33]. ARFID is more common in males and young persons and has a higher co-occurrence with anxiety disorder and neurodevelopmental disorders, including ASD, compared to non-ARFID EDs (AN and BN) [2, 3, 34]. Fisher et al. (2014) reported that 28 of 98 patients with ARFID (28.7%) had a previous history of selective eating since early childhood [3]. Selective eating is one of the problematic eating behaviors and is frequently observed in children with ASD as well.

Eating problems in ASD have also attracted considerable attention since Lucarelli et al. reported a challenging case of ASD with ARFID in 2017 [35]. Eating problems are five times more likely in ASD compared to typically developed (TD) children [36]. In most cases, strong preferences (selective eating) and food refusal are the biggest problems for parents, and around 70% of parents with ASD children express concern over eating problems [37, 38]. Links between the severity of autistic traits and problematic eating behaviors are becoming clearer based on studies investigating autistic traits using the AQ or AQC and problematic eating behaviors using the general Eating Attitudes Test (EAT26) or the children’s version ChEAT26 [39–41]. Significant correlations between total EAT26 and both total AQ and AQ subscales, except for imagination, were found by Christensen et al. [42] Thus, based on these reports, we hypothesize that we can find link between autistic traits and ARFID, and high prevalence of ASD in ARFID, as in AN. However, to our knowledge, there has been no published research examining this association.

The current study investigated the prevalence of ASD and autistic traits in children and adolescents with AN or ARFID. Further, we explored correlations between autistic traits and the severity of FEDs in ED patients recruited from a prospective multicenter cohort study.

## Methods

### Participants

We evaluated the data of 131 children with FEDs from The Japanese Pediatric EDs Outcome: a Prospective Multicenter Cohort Study (J-PED). The J-PED study includes 11 medical institutions throughout Japan. All members of the J-PED study group are board certified by the Japanese Society of Psychiatry and Neurology or the Japan Pediatric Society, have worked with FED patients for more than 6 years, and were trained on the standard structured Mini-International Neuropsychiatric Interview for Children and Adolescents (MINI-KID). Inclusion criteria in the JPED study were: aged under 16 years, having abnormal eating habits and unaccountable weight change. The J-PED members ruled out medical causes for the eating habits and the weight change and checked for any related complications. If the J-PED members suspected that patients had some type of eating disorder, they applied the diagnostic criteria for eating disorders in DSM-5. The JPED study was done to prospectively examine children with FEDs and finished recruitment in 2016, of the children studied, 131 patients with FEDs were recruited. All patients were assessed through direct observation and interview, and diagnoses were guided by the DSM-5 along with the MINI-KID for comorbidity assessment. A developmental history of each patient was obtained by the psychiatrist or pediatrician and psychologists. The exclusion criteria for the J-PED study are self-injury, harmful behavior to others, psychotic symptoms, requirement for constant medical supervision, and age older than 16 years at the first visit. Written informed consent was obtained from all participants and their parents. The 131 ED children were enrolled in the J-PED study from April 2014 to March 2016.

Of the 131 children with ED, 130 were monitored for more than 1 year (one patient withdrew). Each patient’s diagnosis was carefully reviewed within 1 year following their first visit, with discussion among institutes as necessary because FED diagnosis can be challenging and change over time [43]. Of the 130 children with ED, 92 (9–15 years old) were diagnosed with AN (restricting type: 88, binge/purge type: 4), 32 (5–15 years old) with ARFID, and 6 with Unspecified Feeding or Eating Disorder. Consequently, 124 subjects (AN + ARFID) were enrolled in the current study. Patient demographic and physical characteristics are summarized in Table 1 and their familial backgrounds are summarized in supplemental Table 1. All assessment items were performed when they first visit our outpatient clinics (psychiatry or pediatrics).

**Table 1:** Participant characteristics

	HC (*n* = 496)	ED (*n* = 124)	*P*-value
Sex (F: M)	456: 40	114: 10	1.000
Age	13.1 ± 0.8	13.0 ± 1.9	0.272
Height	153.6 ± 6.4	148.9 ± 10.2	**0.000**
Weight	44.5 ± 7.3	30.2 ± 5.6	**0.000**
BMI	18.8 ± 2.6	13.5 ± 1.6	**0.000**
BMI-SDS	−0.4 ± 1.0	−3.5 ± 1.6	**0.000**

*Abbreviations*: *HC* Healthy Control, *ED* Eating Disorders, *BMI* Body Mass Index, *BMI-SDS* Standardised Body Mass Index

To examine differences in autistic traits between FED subjects and a HC population, 496 TD students matched for sex and age were enrolled from among 1366 students attending local junior high schools. Screening of this HC group was conducted using parent and self-report questionnaires asking if they had been diagnosed with any neurodevelopmental or psychiatric disorders and if there were any concerns about developmental issues. Participants with any neurodevelopmental or psychiatric diagnoses and any developmental issues were excluded from the HC group. The demographic and physical characteristics of the HC group are also summarized in Table 1. All assessment items were performed at their school.

### Assessment items

To evaluate the presence and severity of EDs and autistic traits, AQC, body mass index standard deviation scores (BMI-SDS), and ChEAT26 were obtained.

### Autism Spectrum quotient Children’s version (AQC)

The AQC is a parent-reported screening instrument that assesses autistic traits in children between the ages of 6 and 15 years. The original version was published in 2008, and it has since been validated in other countries, including Japan [44, 45]. It contains 50 questions divided into five subscales assessing social skills, attention switching, attention to detail, communication, and imagination. Each question is scored “0” or “1,” and the total AQC is the sum of the 50 questions. Hence, the total AQC ranges between 0 and 50, with higher scores indicating more severe autistic traits. The cut-off for distinguishing ASD in the Japanese version is 25, which means that 85.2% of true ASD cases fall within the range 25 to 50 [44]. In this study, all participants’ parents (mostly mother) completed the AQC, and we calculated the total AQC and five subscale scores. The reliability of the AQC was calculated in our study: Cronbach’s alpha was 0.73 for the total score (50 items), 0.68 for the social skills, 0.51 for the attention switching, 0.55 for the attention to detail, 0.69 for the communication and 0.45 for the imagination.

### Standardized body mass index (BMI-SDS)

The height and weight of both ED and HC children were measured at their first visit to hospital and at school, respectively, and we calculated BMI-SDS. BMI is used to assess body shape. However, absolute BMI is not recommended in assessing underweight children or adolescents as average BMI shows age-related changes in children. Thus, age- and sex-standardized BMI-SDS or BMI percentiles are more accurate indicators of body shape in underweight adolescents. We evaluated associations between autistic traits and BMI-SDS using Pearson’s correlation analysis.

### Children’s eating attitudes test (ChEAT26)

ChEAT26 is a 26-item self-report questionnaire assessing eating attitudes and behavior in children. The adult version was created in 1979, and the child version was created in 1989 [46, 47]. The child version has since been validated in other countries, including Japan [48, 49]. Participants are asked to indicate the ED psychopathology for each item according to a five-point Likert scale ranging from “always” to “never.” The 26 items are divided into five subscales assessing preoccupation with thinness, food preoccupation, dieting, social pressure to eat, and purging. In the Japanese version, possible total score ranges from 0 to 75, with a higher total score indicating more severe eating pathology. The cut-off indicating a possible eating disorder is 18 [48]. The reliability of the ChEAT26 was calculated in our study: Cronbach’s alpha was 0.85 for the total score (26 items), 0.79 for preoccupation with thinness, 0.69 for food preoccupation, 0.72 for dieting, 0.76 for social pressure to eat, and 0.52 for purging.

### Statistical analysis

All data were analyzed using SPSS version 23.0 (IBM Japan, Ltd., Tokyo, Japan). Homogeneity of variance was confirmed with F-test and data normality was tested with Shapiro-Wilk test. The analyses of the quantitative variables among the AN, ARFID, and HC groups were examined by unpaired t-test, Fisher’s exact test, or the Steel-Dwass test. The relations among measures of autistic traits, eating disorder severity, and other psychopathology scales were examined using Pearson correlations. Two tailed *p*-values < 0.05 were considered statistically significant for all tests.

### Ethical considerations

This study was approved by the Ethics Committee of Dokkyo Medical University Saitama Medical Centre (#1336) and by the Medical Ethics Committee of Kurume University School of Medicine (#13211). Written informed consent was obtained from all participants and their parents. The experiments were conducted in accordance with the tenets of the Declaration of Helsinki. The authors declare that they have no conflict of interest.

## Results

The demographic and physical characteristics of the ED and HC group participants are summarized in Table 1. There were no significant differences in sex ratio and mean age between the HC and ED (AN + ARFID) groups. As expected, body weight, BMI, and BMI-SDS were significantly lower in the ED group than in the HC group. Moreover, the ED group was also significantly shorter than the HC group.

The clinical characteristics of the AN and ARFID groups are compared in Table 2. Of the 124 children with ED, 92 were diagnosed with AN and 32 with ARFID. There was no significant difference in age-corrected BMI between the AN and ARFID groups. By contrast, the ARFID group was significantly younger than the AN group. In addition, although females predominated in both groups, there was a greater proportion of males in the ARFID group. The prevalence of ASD was high in both groups, without significant difference (16.3% [15/92] in the AN and 12.5% [4/32] in the ARFID group).

**Table 2:** Comparison of clinical characteristics between AN and ARFID

	AN (*n* = 92)	ARFID (*n* = 32)	*P*-value
Sex (F: M)	89: 3	25: 7	**0.003**
Age	13.4 ± 1.5	11.8 ± 2.4	**0.000**
BMI-SDS	−3.6 ± 1.5	−3.2 ± 1.8	0.175
ChEAT26 total score	24.3 ± 15.1	10.5 ± 6.5	**0.000**
Preoccupation with thinness	4.8 ± 4.8	0.3 ± 0.6	**0.000**
Food preoccupation	4.4 ± 4.2	0.8 ± 1.2	**0.000**
Dieting	8.8 ± 5.9	3.6 ± 2.7	**0.000**
Social pressure to eating	5.9 ± 3.5	5.0 ± 3.8	0.265
Purging	0.4 ± 1.0	0.2 ± 0.5	0.349
Comorbidity of ASD	15/92 (16.3%)	4/32 (12.5%)	0.778

*Abbreviations*: *AN* Anorexia Nervosa, *ARFID* Avoidant/Restrictive Food Intake Disorder, *BMI-SDS* Standardised Body Mass, Index, *ChEAT26* Children’s Eating Attitudes Test, *ASD* Autism Spectrum Disorders

**Fig. 1:**
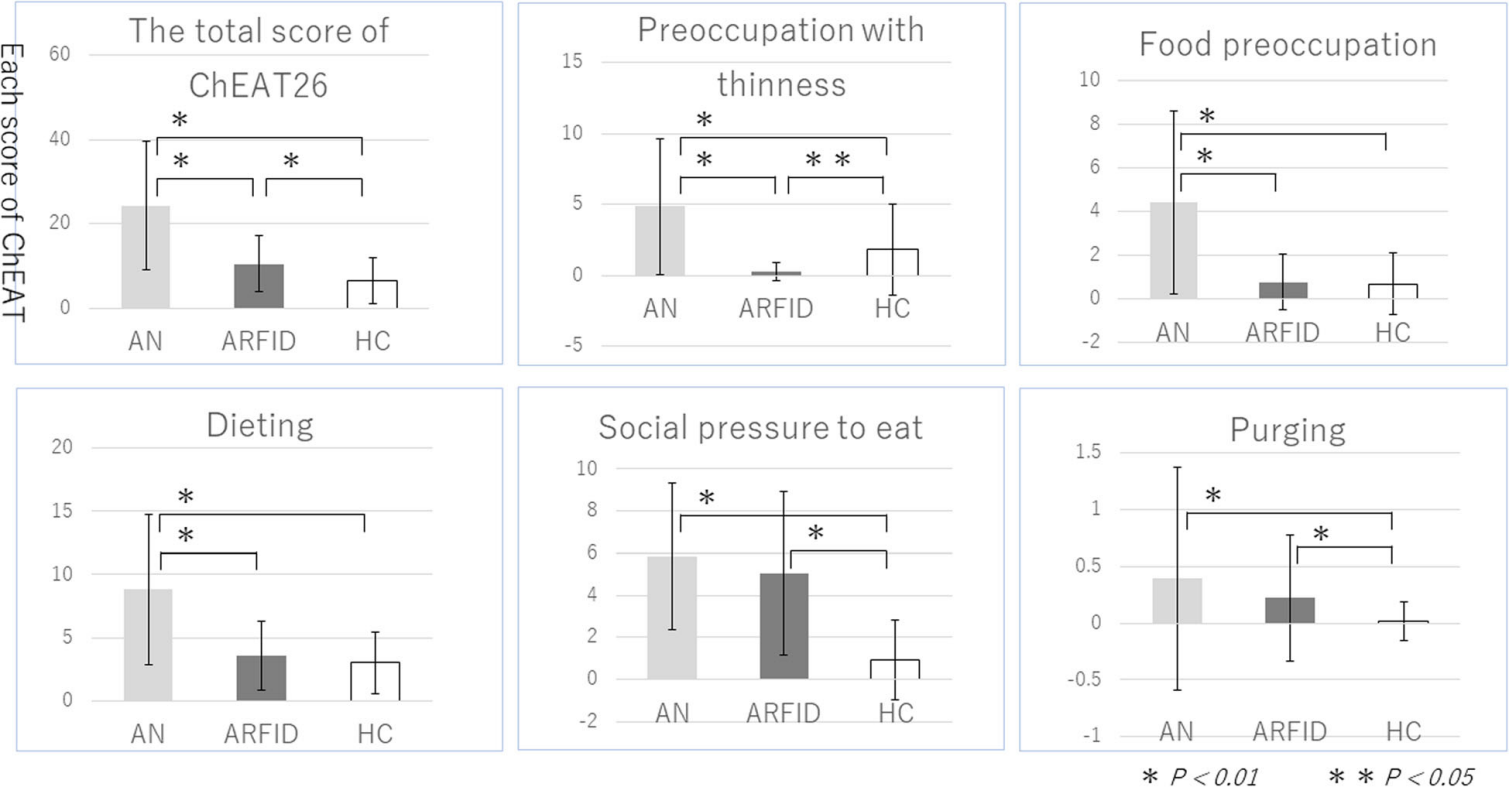
ChEAT26 differences among the AN, ARFID, and HC groups. *Legend*: The ChEAT26 total score was significantly higher in the AN group than in the ARFID group (*p* < .01), and three of the five subscales (preoccupation with thinness, food preoccupation, and dieting) were significantly higher in the AN group than in the ARFID group (*p* < .01)

We analyzed the ChEAT26 data using the Steel-Dwass test. Figure 1 shows ChEAT26 differences among the AN, ARFID and HC groups. The ChEAT26 total score was significantly higher in the AN group than in the ARFID group (*p* < .01), and three of the five subscales (preoccupation with thinness, food preoccupation, and dieting) were significantly higher in the AN group than in the ARFID group (*p* < .01). Total AQC score and all subscale scores were also significantly higher in the AN group than in the HC group (Table 3). Moreover, differences in subscale scores remained after removing AN patients with ASD (*n* = 15), except for that of social skills score, and the total AQC score was still higher in the AN group, but the difference was no longer significant. Alternatively, there were no significant differences in AQC total and subscores between the HC and ARFID groups regardless of ASD diagnosis.

**Table 3:** Comparisons between AN, ARFID and HC groups on AQC scores

	HC			AN			ARFID			*P*-value (vs. control)	
										AN	ARFID
**All patients**											
Number of participants		496			92			32			
Total AQC	15.3	±	6.3	17.0	±	6.8	15.8	±	6.8	**0.020**	0.705
Social skill	3.5	±	2.3	4.1	±	2.3	3.7	±	2.2	**0.031**	0.606
Attention switching	3.0	±	1.9	3.7	±	2.1	3.4	±	2.1	**0.005**	0.268
Attention to detail	3.7	±	1.8	4.2	±	2.0	4.3	±	1.7	**0.019**	0.080
Communication	1.9	±	1.9	2.6	±	2.3	1.9	±	2.1	**0.003**	0.921
Imagination	3.2	±	1.9	2.6	±	1.6	2.7	±	1.5	**0.002**	0.115
**Patients without ASD**											
Number of participants		496			77			28			
Total AQC	15.3	±	6.3	16.9	±	7.0	16.0	±	6.9	0.056	0.602
Social skill	3.5	±	2.3	3.9	±	2.3	4.0	±	2.1	0.138	0.316
Attention switching	3.0	±	1.9	3.6	±	2.1	3.3	±	2.2	**0.011**	0.432
Attention to detail	3.7	±	1.8	4.3	±	1.9	4.3	±	1.7	**0.009**	0.065
Communication	1.9	±	1.9	2.5	±	2.3	1.9	±	2.0	**0.013**	0.983
Imagination	3.2	±	1.9	2.5	±	1.7	2.7	±	1.6	**0.002**	0.195

Notes: mean ± standard deviation (sd)

*Abbreviations*: *AN* Anorexia Nervosa, *ARFID* Avoidant/Restrictive Food Intake Disorder, *HC* Healthy Control, *AQC* Autism spectrum Quotient Children’s version

Associations between autistic traits (AQC total score and subscales) and eating behaviors (BMI-SDS, ChEAT26 total score and subscales) were examined using Pearson correlation analysis (Table 4). In the AN group, there were no significant correlations between AQC scores (total and subscale scores) and BMI-SDS, ChEAT26 total score, and ChEAT26subscales. In the ARFID group, however, there were significant correlations between total AQC and total ChEAT26 (*p* = .038), total AQC and ChEAT26 subscales food preoccupation (*p* = .038) and social pressure to eat (*p* = .008), AQC subscale social skill and ChEAT26 subscale preoccupation with thinness (*p* = .009), AQC subscale attention switching and ChEAT26 subscale food preoccupation (*p* = .026), AQC subscale attention to detail and total ChEAT26 (*p* = .039), AQC subscale attention to detail and ChEAT26 subscale social pressure to eat (*p* = .030), and AQC subscale imagination and ChEAT26 subscale social pressure to eat (*p* = .035). Moreover, some correlations between AQC and ChEAT26 scores remained after removing ARFID patients with ASD (supplemental Table 2).

**Table 4:** Correlation between BMI-SDS, ChEAT26 and AQC

	Total AQC		Social skill		Attention switching		Attention to detail		Communication		Imagination	
	CC:r	*P*-value	CC:r	*P*-value	CC:r	*P*-value	CC:r	*P*-value	CC:r	*P*-value	CC:r	*P*-value
**AN (*n*** **= 92)**												
BMI-SDS	0.035	0.743	0.138	0.193	0.034	0.751	−0.164	0.123	0.090	0.398	−0.022	0.839
ChEAT26	−0.039	0.717	−0.169	0.111	0.101	0.344	0.046	0.670	0.028	0.796	−0.150	0.157
Preoccupation with thinness	− 0.063	0.553	− 0.137	0.199	0.051	0.630	0.008	0.939	−0.019	0.859	−0.126	0.238
Food preoccupation	−0.021	0.846	−0.119	0.262	0.099	0.355	0.036	0.733	0.029	0.789	−0.132	0.215
Dieting	−0.061	0.567	−0.165	0.120	0.066	0.539	0.035	0.747	0.021	0.847	−0.184	0.084
Social pressure to eating	0.068	0.523	−0.083	0.439	0.140	0.188	0.109	0.305	0.071	0.506	−0.006	0.959
Purging	−0.101	0.345	−0.158	0.138	0.019	0.857	−0.136	0.201	−0.016	0.884	−0.042	0.692
**ARFID (*n*** **= 32)**												
BMI-SDS	−0.081	0.678	−0.146	0.443	−0.059	0.756	−0.141	0.458	−0.129	0.498	0.148	0.434
ChEAT26	0.395	**0.038**	0.180	0.350	0.345	0.067	0.385	**0.039**	0.202	0.294	0.282	0.139
Preoccupation with thinness	0.239	0.213	0.466	**0.009**	0.014	0.944	−0.197	0.297	0.141	0.458	0.304	0.103
Food preoccupation	0.388	**0.038**	0.117	0.539	0.406	**0.026**	0.239	0.203	0.332	0.073	0.137	0.472
Dieting	0.097	0.616	−0.075	0.696	0.142	0.454	0.239	0.203	0.060	0.752	0.065	0.733
Social pressure to eating	0.481	**0.008**	0.358	0.052	0.296	0.112	0.396	**0.030**	0.190	0.316	0.387	**0.035**
Purging	0.267	0.162	0.188	0.319	0.170	0.369	0.149	0.431	0.250	0.183	0.250	0.183

*Abbreviations*: *BMI-SDS* Standardised Body Mass Index, *ChEAT26* Children’s Eating Attitudes Test, *AQC* Autism spectrum Quotient Children’s version, *CC* correlation coefficient

## Discussion

We evaluated the prevalence of ASD and autistic traits in children and adolescents with AN (*n* = 92) or ARFID (*n* = 32). Among the FED group, there were no BN cases possibly, because all participants were children and we excluded patients demonstrating self-injury and harmful behavior to others. Differences in clinicodemographic characteristics between the AN and ARFID groups were in accordance with previous reports [2, 3, 48]. Specifically, the ARFID group exhibited a higher proportion of males and younger patients, total ChEAT26 score was lower than that in the AN group, and there was no difference in age-corrected BMI (Table 2). Collectively, these similarities support the reliability of the diagnostic processes used in this study.

The first case study of ASD–AN comorbidity, describing the difficulties in managing a 12-year-old female with AN, mild intellectual disability, and ASD, was reported in 1980 [50]. A possible link between ASD and AN was first proposed by Gillberg in 1983, who reported the case of a female with AN and three autistic cousins [6]. Gillberg and Rastam (1992) investigated 51 adolescents with AN and reported that one male had Asperger’s spectrum disorder, three females had histories suggesting high functioning autism, and 13 had social interaction problems in childhood [51]. These results suggest that AN and ASD patients share preoccupation, rituals, and social interaction deficits [51]. Inspired by these early reports, numerous larger-scale studies on the prevalence of ASD in FEDs have been conducted over the past three decades. A systematic review by Huke et al. (2013) reported that 22.9% of AN patients across multiple studies also have ASD (range 8 to 37%). However, most of these were adult populations, and fewer studies have investigated AN–ASD comorbidity in children and adolescents. The reported prevalence of ASD is generally lower in young FED patients compared to adults, with various studies reporting only 1 in 22 AN patients (age range: 10–16) meeting ICD-10 criteria for ASD (4.5%) [52], 6 in 150 AN patients (age range: 12–21) with possible or definite ASD (4%) [53], and 4 in 40 AN patients (age range: 12–18) diagnosed with ASD (10%) [54]. In the present study, the ASD prevalence in AN was higher at 16.3% (15 in 92). As patients currently ill with AN tend to show more severe autistic traits due to starvation [55], it is important to obtain an early developmental history to diagnose ASD. Here, we applied the standard structured diagnostic interview to patients and parents to construct an early developmental history, and diagnosis was carefully reviewed within 1 year after first hospital visit. One potential reason for the higher prevalence of ASD in AN in this study is selection bias as all 11 institutes in the J-PED study are primary regional medical centers, so the ED cases are often complex and difficult to treat. Therefore, there is some possibility that ED patients with ASD were selected because of referral or treatment failure at local hospitals. Indeed, ED patients with elevated autistic traits tend to have a higher rate of hospitalization, and symptoms associated with ASD are associated with lower FED treatment efficacy [19, 56].

We also found a high prevalence of ASD in ARFID patients (12.5%, 4/32). As ARFID is newly classified in DSM-5, there are few investigations on this disorder. Nicely et al. (2014) retrospectively reviewed 173 patients between 7 and 17 years old admitted to a day program for younger patients with ED and found that 39 (22.5%) met the DSM-5 criteria for ARFID. These patients were less likely to demonstrate typical ED characteristics, being more common in males and in young persons and lower total ChEAT26 scores. These clinical features corresponded with our results (Table 2), however, interestingly, the prevalence rate of ASD was much higher in their study: 33.3% (13/39). A possible reason why there are differences of prevalence is because of racial differences between their study and ours that may have influenced it.

Another aim of the current study was to investigate autistic traits in children with AN or ARFID. Autistic traits in AN, such as social impairments and obsessive–compulsive traits, are garnering substantial research attention as they suggest shared pathomechanisms. An overlap between ASD traits and AN has been demonstrated using multiple psychometric tools. For instance, Hambrook et al. (2008) reported a significantly higher total AQ score and three subscale scores (social skills, attention switching, and imagination) in 22 women with AN [26]. Their results and those reported here are in accordance with neuropsychological studies investigating cognitive processing dysfunction in AN related to theory of mind [9, 10] and set shifting [18]. Some of these neuropsychological deficits are more conspicuous among patients with a prolonged course of AN compared to shorter illness [57]. According to a recent systematic review of ASD in AN by Westwood et al. (2016), total AQ and AQC scores and four of the five subscales (social skills, attention switching, communication, and imagination) were significantly higher in AN patients [28]. In the current study, AQC scores were significantly higher in AN compared to HCs. These high autistic traits in Japanese AN patients correspond with those of previous studies, which suggests that these associations are independent of cultural attitudes.

Autistic traits in AN have also been examined using alternative instruments such as the Developmental, Dimensional and Diagnostic Interview, short version (3Di-sv) [52, 54], Autism Diagnostic Observation Schedule 2nd edition (ADOS-2) [54, 55, 58, 59], and Development and Well-being Assessment (DAWBA) [53], all of which found higher autistic traits in AN compared to HCs. Westwood et al. (2017) reported that 14 of 60 females with AN (23.3%) scored above the ASD cut-off on ADOS-2, of which 7 displayed speech abnormalities associated with ASD and 4 repetitive behaviors or excessive interest in unusual or highly specific topics and objects [55]. In this study, we did not apply other instruments assessing autistic traits apart from AQC. Future studies should adopt these instruments as well.

To our knowledge, only two studies have investigated correlations between autistic traits and the severity of eating disorder symptoms, both in adults. Tchanturia and colleagues (2013) reported that a greater proportion of AN patients scored above the ASD cut-off on the AQ short version AQ-10 compared to controls [60]. However, autistic characteristics were not significantly correlated with either total scores or subscale scores on the Eating Disorder Examination Questionnaire, a 36-item self-report measure of eating disorder symptomatology and behaviors. Calderoni et al. (2015) also reported no significant correlations between autistic traits and scores on the EAT26, except for the communication subscale [61]. In the current study, there were also no significant correlations between AQC scores and BMI-SDS, total ChEAT26, or ChEAT26 subscales among the AN group, thus confirming these previous results and extending these findings to a pediatric population. These results may suggest that autistic traits are not linked with exacerbation of AN but rather to onset and its maintenance.

Since ARFID is a newly listed diagnosis in DSM-5, there are only a few reports on associations with autistic traits. Nicely et al. (2014) reported a high rate of ASD comorbidity in ARFID patients, consistent with the current results [2]. They also found that many ARFID patients had a history of selective eating in early childhood, which is also observed as a problematic eating behavior in children with ASD. On the basis of these results, we hypothesized that young ARFID patients would demonstrate higher autistic traits as observed in AN. However, the ARFID group did not exhibit significantly higher AQC total and subscale scores compared to the HC group. There is possibility that the AQC scores of HC in our study may be high because we recruited them only from an urban area [48]. Alternatively, statistically significant correlations were found between total AQC and total ChEAT26 scores as well as between certain AQC and ChEAT26 subscales (Table 4). These results may suggest that autistic traits are not linked to ARFID onset and maintenance but rather to exacerbation. Some correlations in these results are clinically acceptable; for instance, food preoccupation in ARFID was correlated with attention switching difficulties, which can be interpreted that children with ARFID are sometimes seized with fear of vomiting or food choking, in addition that they cannot get such thoughts out of their mind. These findings are also consistent with the correlations between EAT26 and AQ in adults with ASD [39–41], although the correlations among subscales differed. For example, Carton et al. (2014) reported that food preoccupation was correlated with social skills, communication, and attention switching, whereas the current study found a correlation between food preoccupation and attention switching. Therefore, the specific associations may differ between adults and children. Recently, several studies have reported phenotypic heterogeneity among ARFID patients, so the boundaries of ARFID require further research, especially regarding the three-dimensional model of ASD encompassing lack of interest in food, selective eating founded on sensory sensitivity, and fear of unpleasant consequences [33, 62]. Variable operationalization of criteria based on the three-dimensional model may thus alter the specific associations between autistic traits and ED symptoms.

The current study has several limitations. First, the numbers of AN and ARFID patients differed as they were selected from a prospective cohort, which may have introduced a sample size effect. However, the prospective study design and patient recruitment method may also be viewed as strengths. Prospective studies have better epidemiological validity as recruitment is based on presentation to a healthcare institute without self-selection. Second, we did not use a gold standard diagnostic instrument such as ADOS-2 or Autism Diagnostic Interview-Revised since we had to match the diagnostic measures among 11 institutions. Instead, we applied a standard structured diagnostic interview (MINI-KID), and DSM-5 diagnosis was guided by direct observation and interviews as well as developmental history. To assess the psychopathology of individuals with autistic traits, long-term direct observation is the best way, considering this, there is room for improvement. Third, this study was conducted in Japan, and all participants were Japanese. Thus, our results are typical of a homogeneous society with little diversity in race, culture, religion, and education. Nonetheless, similarities with findings in North America and Europe are suggestive that these associations between FEDs and ASD reflect basic neurodevelopment processes with little influence of culture or genetic background. On the other hand, no study has examined the impact of industrialization on the relation between FEDs and autistic traits. Change of industrial structures and development of the service industry affect people with poor social interaction skills. Therefore, we need to investigate it in developing countries as well.

In conclusion, ASD prevalence is high in Japanese children with AN or ARFID (16.3 and 12.5%, respectively). The AN group exhibited significantly higher total AQC and all subscale scores, however there were no significant correlations between AQC and ChEAT26. Alternatively, we newly reported that AQC scores did not differ between ARFID and controls, but there were multiple correlations between various AQC and ChEAT26 scores. To our knowledge, the present study is the first to assess autistic traits in patients with ARFID. FED patients with high autistic traits are associated with lower treatment efficacy, therefore, to understand the features of autistic traits in FEDs will be key to clinical treatment. For example, if the patient is cognitively compromised due to autistic traits such as limited set shifting and cognitive inflexibility, nutrition education and explanation of a meal plan should be brief and simple, and sometimes provided using visually attractive leaflets for better understanding.

## Supplementary Information

**Additional file 1: Supplemental Table 1.** Parents’ background.

**Additional file 2: Supplemental Table 2.** Correlation between BMI-SDS, ChEAT26 and AQC.

## Data Availability

The datasets analyzed in this study are available from the corresponding author upon reasonable request.
